# The characteristic of the synonymous codon usage and phylogenetic analysis of hepatitis B virus

**DOI:** 10.1007/s13258-020-00932-w

**Published:** 2020-05-27

**Authors:** Xiaoming Qi, Chaojun Wei, Yonghong Li, Yu Wu, Hui Xu, Rui Guo, Yanjuan Jia, Zhenhao Li, Zhenhong Wei, Wanxia Wang, Jing Jia, Yuanting Li, Anqi Wang, Xiaoling Gao

**Affiliations:** 1 The Institute of Clinical Research and Translational Medicine, Gansu Provincial Hospital, 204 Donggang West Road, Chengguan District, Lanzhou, 730000 China; 2 NHC Key Laboratory of Diagnosis and Therapy of Gastrointestinal Tumor, Gansu Provincial Hospital, Lanzhou, 730000 China; 3 Gansu Provincial Biobank and Bioinformation Engineering Research Center, Lanzhou, 730000 China

**Keywords:** Hepatitis B virus, Codon usage pattern, Phylogenetic evolution, Mutation pressure, Translation selection

## Abstract

**Background:**

Hepatitis B virus (HBV) infection is a crucial medical issue worldwide. The dependence of HBV replication on host cell machineries and their co-evolutionary interactions prompt the codon usage pattern of viral genes to translation selection and mutation pressure.

**Objective:**

The evolutionary characteristics of HBV and the natural selection effects of the human genome on the codon usage characteristics were analyzed to provide a basis for medication development for HBV infection.

**Methods:**

The codon usage pattern of sequences from different HBV genotypes of our isolates and reference HBV genome sequences downloaded from the National Center for Biotechnology Information (NCBI) database were analyzed by computing the relative synonymous codon usage (RSCU), nucleotide content, codon adaptation index (CAI) and the effective number of codons (ENC).

**Results:**

The highest ENC values were observed in the C genotypes, followed by the B genotypes. The ENC values indicated a weak codon usage bias (CUB) in HBV genome. The number of codons differentially used between the three genotypes was markedly higher than that of similarly used codons. High CAI values indicated a good adaptability of HBV to its host. The ENC plot indicated the occurrence of mutational pressure in the three genotypes. The mean Ka/Ks ratios in the three genotypes were lower than 1, which indicated a negative selection pressure. The CAI and GC3% plot indicated the existence of CUB in the HBV genome.

**Conclusions:**

Nucleotide composition, mutation bias, negative selection and mutational pressure are key factors influencing the CUB and phylogenetic diversity in HBV genotypes. The data provided here could be useful for developing drugs for HBV infection.

**Electronic supplementary material:**

The online version of this article (10.1007/s13258-020-00932-w) contains supplementary material, which is available to authorized users.

## Introduction

Hepatitis B virus (HBV) infection, the main causal factor for liver diseases such hepatitis, cirrhosis and liver cancer, is a significant global health concern worldwide (Benhenda et al. 2013; Binh et al. 2019; Bonvicino et al. 2014; Kim et al. 2007, 2017; Sarkar and Chakravarty 2015; Shih et al. 2018). The incidence of HBV is annually increasing, and developing suitable therapeutic approaches is extremely difficult (Li et al. 2017; Nelson et al. 2016; Stasi et al. 2016). HBV is a small particle retroid virus characterized by its circular partially double-stranded DNA genome (Gerelsaikhan et al. 1996), with four over-lapping open reading frames including large S region, PreC/C, X, P gene (Kramvis and Kew 1998; Ma et al. 2015).

The large S protein is present in external (Le-HBsAg) and internal (Li-HBsAg) topological conformations (Churin et al. 2015). The Le-HBsAg conformation allows the attachment of HBV to cellular receptors, which is the initial step of viral infection. In Li-HBsAg conformation, the large S protein participates in virion morphogenesis and regulates the contact with the nucleocapsid (Taylor 2013). The surface protein gene S encoding for large, middle and small S proteins (De Maddalena et al. 2007) is important for studying genome evolution (Chen et al. 2013) because of its complete overlapping with the polymerase gene (Pavesi 2015; Torresi 2002). The HBV genome evolution depends on the complex interaction between viral and host factors, which determines the persistence and progression of HBV infection. Phylogenetic studies of HBV genome have revealed at least ten HBV genotypes, designated A–J (Tian and Jia 2016). These genotypes are major phylogenetic variants playing certain roles in the pathogenesis of HBV infection and are correlated with the progression and long-term outcome of HBV and its epidemiology, which indicates their role in clinical practice (Lin and Kao 2017; Tian and Jia 2016). Generally, genotype A is associated with a better response to interferon therapy; genotype C and, to lesser extent, B usually represent a risk factor for perinatal infection and are associated with advanced liver conditions such as cirrhosis and HCC (Lee et al. 2013; Yang et al. 2008); genotype D may be linked with poor response to interferon therapy (Tian and Jia 2016).

HBV genotypes are distributed in an ethnogeographical manner. This is caused by genome evolution secondary to natural selection, mutational pressure and genetic drift (Guan et al. 2018; Kattoor et al. 2015; Li et al. 2019; Tyagi et al. 2017). Codon usage bias, the preferential use of codons encoding a given amino acid, is vital for examining the adaptation of exogenous genes to the hosts and can play a significant role in enhancing the expression of these genes via codon optimization (Goni et al. 2012; Kattoor et al. 2015; Li et al. 2019; Zhou et al. 2013). Codon usage is equally important for studying the evolution and ecological adaptation of diverse organisms (Chakraborty et al. 2017; Ma et al. 2016; Muthabathula et al. 2018; Zhou et al. 2014). The evolutionary characteristics of HBV and the natural selection effects of the human genome on the codon usage characteristics is important for providing a basis for precision medicine. Therefore, studies on the codon usage pattern of HBV would be vital in elucidating the evolution of HBV, its adaptation to the host and the molecular mechanism in hepatitis, and provide useful data on the virulence of each HBV genotype. However, studies on the CUB of HBV are scarce (Li et al. 2015a; Ma et al. 2011; Pavesi 2015), which needs further in-depth analysis.

In our study, genotyping of HBV sequences from patients in Gansu province was performed and the synonymous codon usage pattern and evolutionary dynamics of different HBV genotypes as well as the adaptation of HBV and its host. The findings of this work will be essential in elucidating the mechanisms driving the molecular evolution of HBV and hepatitis B pathogenesis, while providing a theoretical basis for clinical practice.

## Methods

### Study population

A total of 79 patients (47 men and 32 women; mean age = 39.57 ± 13.78 years old, range 13–75) were included in this study. The patients visited Gansu Provincial Hospital between November 2017 and January 2018. All patients had persistent seropositivity of HBsAg and showed: (1) no evidence of hepatocellular carcinoma (HCC) or other metastatic liver disease and (2) no evidence for concomitant hepatitis HCV/HDV or HIV infection or autoimmune liver disease. Our study was reviewed and approved by the institutional Ethics Committee of Gansu Provincial People Hospital. All procedures performed in studies involving human participants were in accordance with the ethical standards of the institutional research committee of Gansu Provincial People Hospital and complied with the 1964 Helsinki declaration and its later amendments or comparable ethical standards.

### Demographic, biochemical and serologic data

HBsAg, anti-HBs, HBeAg, anti-HBe, anti-HCV, and anti-HIV were determined by the microparticle enzyme immunoassay method while anti-HBV was detected by the enzyme immunoassay method (Abbott Laboratories, IL). HBV DNA levels were tested by using a commercial liquid hybridization assay (Digene, MD), with a lower limit of detection of 5 pg/ml.

### HBV DNA extraction and quantification

HBV DNA was isolated from the patient plasma following the procedure provided by the manufacturer using “Instant Virus DNA kit” (AJ Roboscreen, Analytikajena Biosolutions, GmbH, Germany). The DNA was quantified following the manufacturer’s recommendations using the RoboGene^®^ HBV Quantification kit (AJ Roboscreen, analytikajena Biosolutions, GmbH, Germany).

### HBV genotyping

The genotype specific primers were used for genotyping by PCR. The regular PCR reaction mixture was as follows: universal primers S1-2 and P1A (1 µl each), 10 µl GoTaq^®^ Green Master mix (Promega, USA), template DNA (6 µl) and 2 µl of ddH_2_O. The reaction program was as follows: 95 °C for 10 min, 30 cycles of 20 s at 94 °C, 20 s at 55 °C and 1 min at 72 °C, followed by 7 min at 72 °C on an ABI 9700 PCR platform (USA). For nested PCR, Mix 1 with a common antisense primer and sense primers for A, B and C genotypes and mix two with antisense primers for D, E and F genotypes with a common sense primer were used. After 1st round of PCR, 2 µl of the product were taken and added as DNA template to each mix; the reaction mixture was composed of 1 µl of each primer, 8 µl of ddH2O and 8 µl of GoTaq^®^ Green Master mix. The nested PCR conditions were as follows: 10 min at 95 °C, followed by 40 cycles of 45 s at 94 °C, 20 s at 63 °C, and 60 s at 72 °C, followed by 7 min at 72 °C with the above amplification platform. The PCR products were separated on 2% agarose gel and a solution of ethidium bromide was used for staining. The revelation was done by ultraviolet fluorescence (BioRad Gel Doc-XR, USA). Samples with viral load higher than 100 IU/ml but whose genotypes were not identified or detected were considered as untypable samples.

### DNA sequencing data and phylogenetic analysis

Twenty-one HBV nucleotide sequences were downloaded from Genbank in the National Center for Biotechnology Information (NCBI) database (http://www.ncbi.nlm.nih.gov/Genbank/) and added to our sequencing data for analysis. The nucleotide composition percentage (A%, U%, G% and C%) and the percentage of nucleotide in the third position of the codon (A3%, U3%, G3% and C3%) of HBV coding sequence were computed using the CAIcal platform (http://genomes.urv.es/CAIcal) (Puigbò et al. 2008). The Mega6 software (Tamura et al. 2013) was used for the construction of the neighbor joining phylogenetic tree by setting the bootstrap value to 1000.

### The calculation of the relative synonymous codon usage (RSCU)

The RSCU values for the coding sequences of our 79 HBV and 21 reference HBV sequences were computed using the CAIcal platform (http://genomes.urv.es/CAIcal) (Puigbò et al. 2008). RSCU values equal to 1.0 indicated that the codon was selected equally and randomly whereas the RSCU higher or lower than 1.0 implied higher frequency or lower frequency, respectively. In addition, the codons with RSCU exceeding 1.6 were considered as over-represented synonymous codons while those with RSCU lower than 0.6 under-represented ones.

### ENc analysis

ENc analysis, which quantifies the absolute CUB, was used to estimate the CUB of the coding sequences of HBV. ENc = 20 indicates an excessive CUB while EN_C_ = 61 indicates that there is no CUB. Smaller ENc values indicate larger codon preference in a gene. ENC is calculated using the formula: Nc = 2 + 9/F2 + 1/F3 + 5/F4 + 3/F6, wherein the Fk (k = 2, 3, 4, or 6) represents the mean of the Fk values for k-fold degenerate amino acids. The F value is the probability to randomly choose two identical codons encoding for an amino acid. Herein, the ENc values were generated using the CodonW software version 1.4.4.

### Principal component analysis PCA

Principal component analysis (PCA) was performed to uncover the main tendency of the codon usage pattern between HBV strains. The PCA was done using the R package “ade” based on the RSCU values.

### Codon adaptation index (CAI) analysis

To explore the codon usage preferences, we analyzed the codon adaptation index using the online tool CAIcal (CAI; http://genomes.urv.es/CAIcal) (Puigbò et al. 2008) considering *H. sapiens* cells as reference. Human gene datasets were arbitrarily chosen from the Ensembl (http://www.ensembl.org) database. Student’s t test was applied to analyze the difference among CAI values from different groups. The expected value of CAI (e-CAI) was computed at the 95% confidence interval online using the CAIcal tool (http://genomes.urv.es/CAIcal/E-CA) based on the Kolmogorov–Smirnov test. The RSCU values of the host *H. sapiens* were downloaded from the codon usage database (https://hive.biochemistry.gwu.edu/dna.cgi?cmd=refseq_processor&id=545408).

### Correlation analysis

The Pearson’s correlation was performed to unveil the correlation between variables using the Hmisc package (https://cran.r-project.org/web/packages/Hmisc/index.html) in R. The correlation between nucleotide composition in the third position of codon (A_3_%, C_3_%, U_3_% and G_3_%) and general nucleotide composition (A%, C%, U% G%) in HBV coding sequences was determined. The correlation between the codon usage pattern and A_3_%, C_3_%, U_3_% and G_3_% of HBV was also computed.

### Statistical analysis

The differences between groups were analyzed using the GraphPad Prism software. One-way analysis of variance (one-way ANOVA) was performed and followed by the Bonferroni post-hoc test. p value cutoff lower than 0.05 was considered for statistical differences between groups.

## Results and discussion

### The cluster of HBV sequences from patients and reference sequence by Phylogenetic analysis

**Fig. 1:**
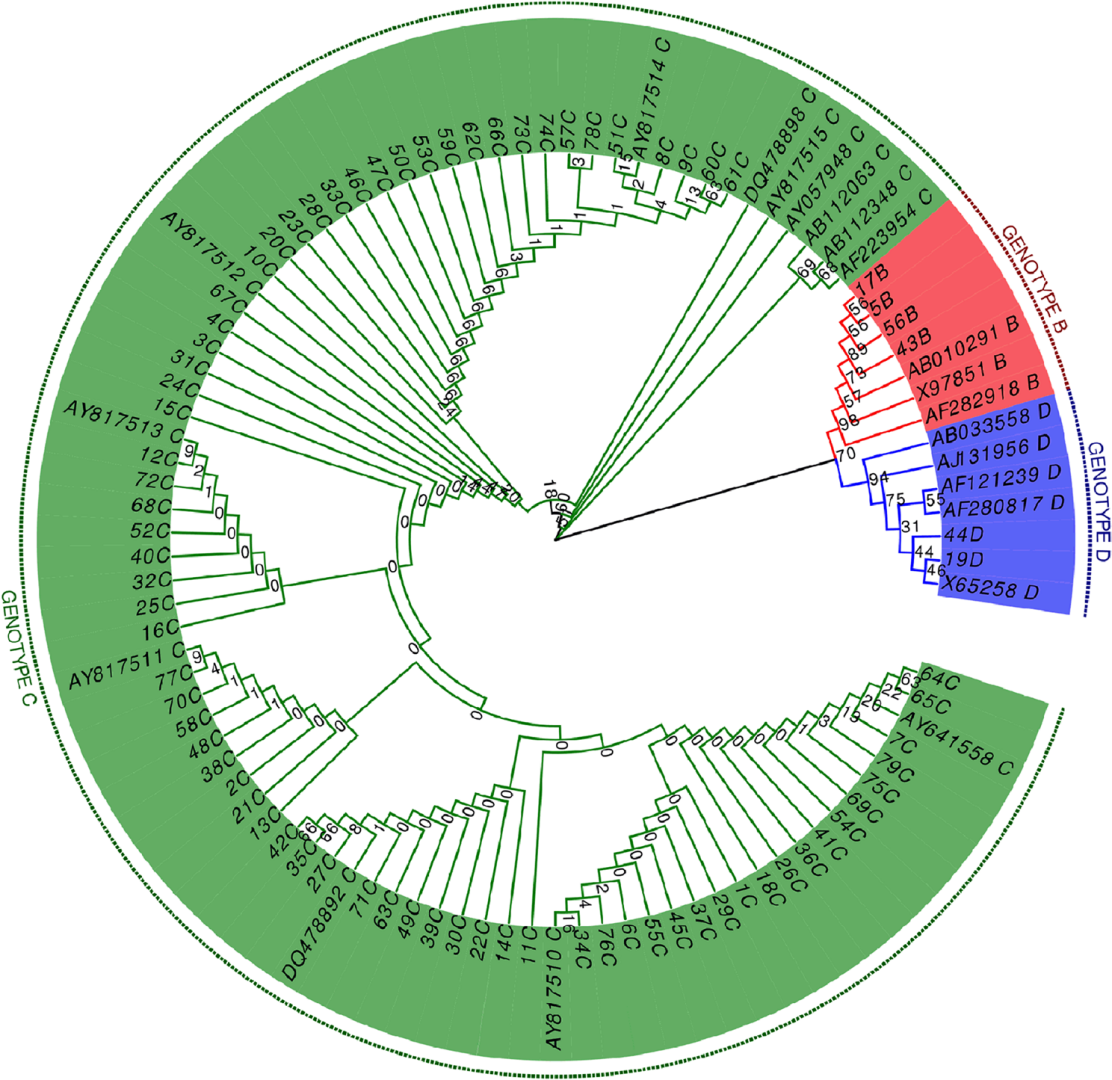
Phylogenetic tree of HBV sequence from our patients and reference sequence. HBV isolated from the serum of chronic HBV infected patients visited Gansu Provincial Hospital. Phylogenetic tree was constructed by Neighbor-Joining method, based on genotype sequences of 79 HBV isolates and 21 reference sequences from GenBank database

To identify the genotypes of HBV that infected the patients, PCR genotyping was performed and followed by sequencing. Our data showed that three different genotypes of HBV (B, C and D genotypes) were identified from the HBV patients. Neighbor-Joining phylogenetic tree analysis showed that there were few substitutions in the sequences. They could be distinctly clustered to the reference sequences obtained from the NCBI database (Fig. 1). There was a significant difference in HBV sequences from different genotypes despite their high identity (Fig. 1). Different sequences of the same genotype were clustered together and had good aggregation with the reference sequences of the same genotype (Fig. 1).

### Nucleotide composition analysis of HBV

**Fig. 2:**
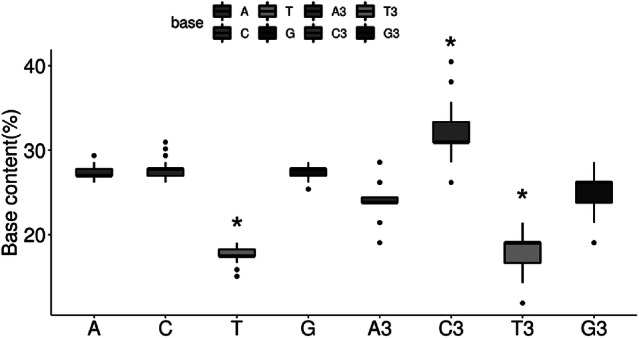
Base content and composition in bases at third position of codons in HBV sequences. *p < 0.05 compared to A or A3 content as revealed by one-way ANOVA analysis,

**Fig. 3:**
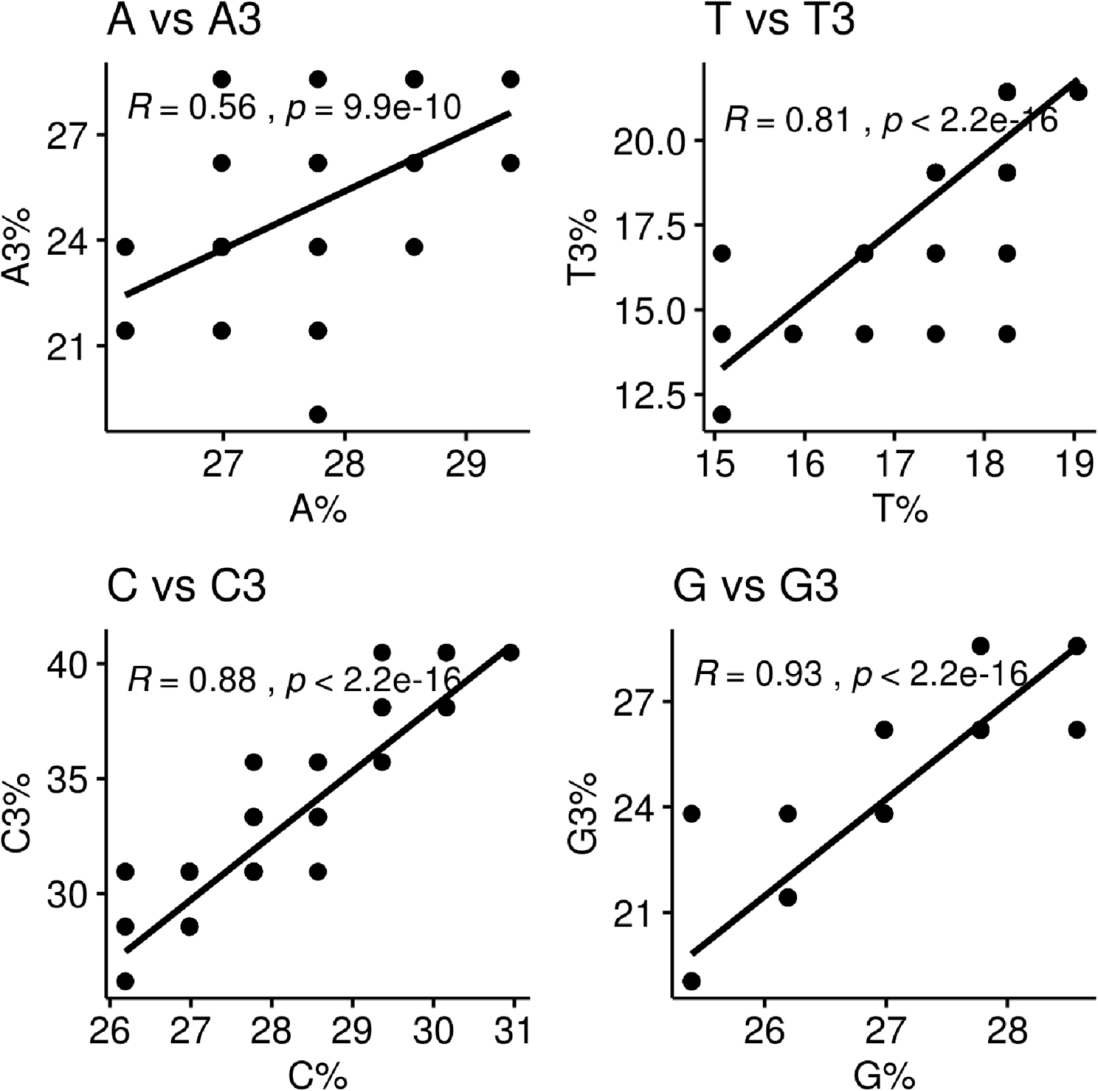
The overlap scatter-plot of the content of each nucleotide and the content of each nucleotide at the synonymous third position of codon

CUB can be greatly influenced by the overall nucleotide content of the genome (Li et al. 2015b; Ma et al. 2015). A previous study suggested that nucleotide bias is an important factor of the virus-specific codon usage that limits the role of codon selection and translational control (van Hemert and Berkhout 2016; van Hemert et al. 2016). Therefore, we first determined the nucleotide compositions of the HBV genome to highlight the potential influence of the nucleotide constraints on codon usage. Our results indicated that the mean compositions of nucleotides A (27.31% ± 0.67), C (27.75%±0.95) and G (27.44% ± 0.80) were significantly higher compared to T (17.50% ± 0.87) (Fig. 2). No significant difference (p > 0.05) was found among A, C and G composition (Fig. 2). One-way ANOVA test indicated that the differences in A vs. T, C vs. T, and T vs. G comparisons were statistically significant (p < 0.05). The percentages of nucleotides at the third codon position were: 24.26% ± 1.94 for A3; 31.83%±3.00 for C3; 18.48% ± 2.30 for T3; and 25.43% ± 2.37 for G3 (Fig. 2). These values were different from the expected total nucleotide contents (the percentage of a given nucleotide in the analyzed sequences). Specifically, the percentages of A3, C3, T3 and G3 were significantly lower than A, C, T and G. The Pearson correlations among nucleotide content (A%, T%, G%, C%, GC%) and the percentage of nucleotides at the third position of the codon (A3%, T3%, G3%, C3%, GC3%) were analyzed to clarify whether the effect on CUB was due to translational selection or pressure alone. The overlap scatter-plot of the content of each nucleotide and the content of the nucleotide at the third codon position was depicted in Fig. 3. A positive correlation was generally indicated between the nucleotide composition and the nucleotide composition of the nucleotide at the third codon position in each of the different genotypes of HBV (Fig. 3). The positive correlations between A% and A3% (r = 0.56, p < 0.001) and C% and C3% (r = 0.88, p < 0.001) suggested that the constraint of nucleotide content under mutation pressure defines the profile of CUB. In addition, significant positive correlations were found between T% and T3% (r = 0.81, p < 0.001) (Fig. 3), suggesting that natural selection might not impact on the codon usage pattern.

### Codon usage patterns in HBV genotypes

**Fig. 4:**
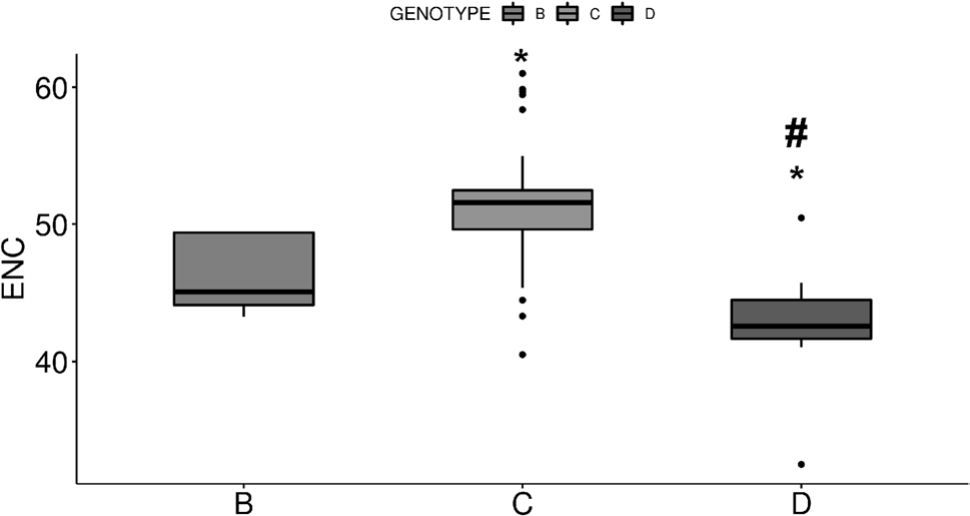
The ENC values of different codons in the sequences of different HBV sequences. *p < 0.05 compared to B, ^#^p < 0.05 compared to C

To assess the overall CUB in HBV, the extent of CUB in the HBV genotypes was determined and compared based on the effective number of codons (ENC) and relative synonymous codon usage (RSCU) values of the 79 HBV sequences codon and CAIcal, respectively. According to previous studies, ENC values less than 35 mean high codon preference and ENC values more than 50 reveal general random codon usage (Wang et al. 2018). Our data showed that the ENC mean values (Fig. 4) ranged from 32.5 to 61, with an average ENC value of 50.23 ± 4.25 considering all three genotypes. The ENC value indicated a relatively weak CUB in HBV sequences because only 1% (D genotype) of the HBV sequences had an ENC value < 35. The mean ENC value of HBV genotype C (51.12 ± 3.44) was significantly (p < 0.05) higher than those of B (46.39 ± 2.88) and D (42.35 ± 5.86). Significant (p < 0.05) difference in ENC was also found between B and D genotypes. Thus, the genotype C tended to use more types of codons to produce proteins, suggesting that their genes might undergo weaker selection constraints with respect to replication speed and transcription efficiency and accuracy compared to B and D genotypes.

**Fig. 5:**
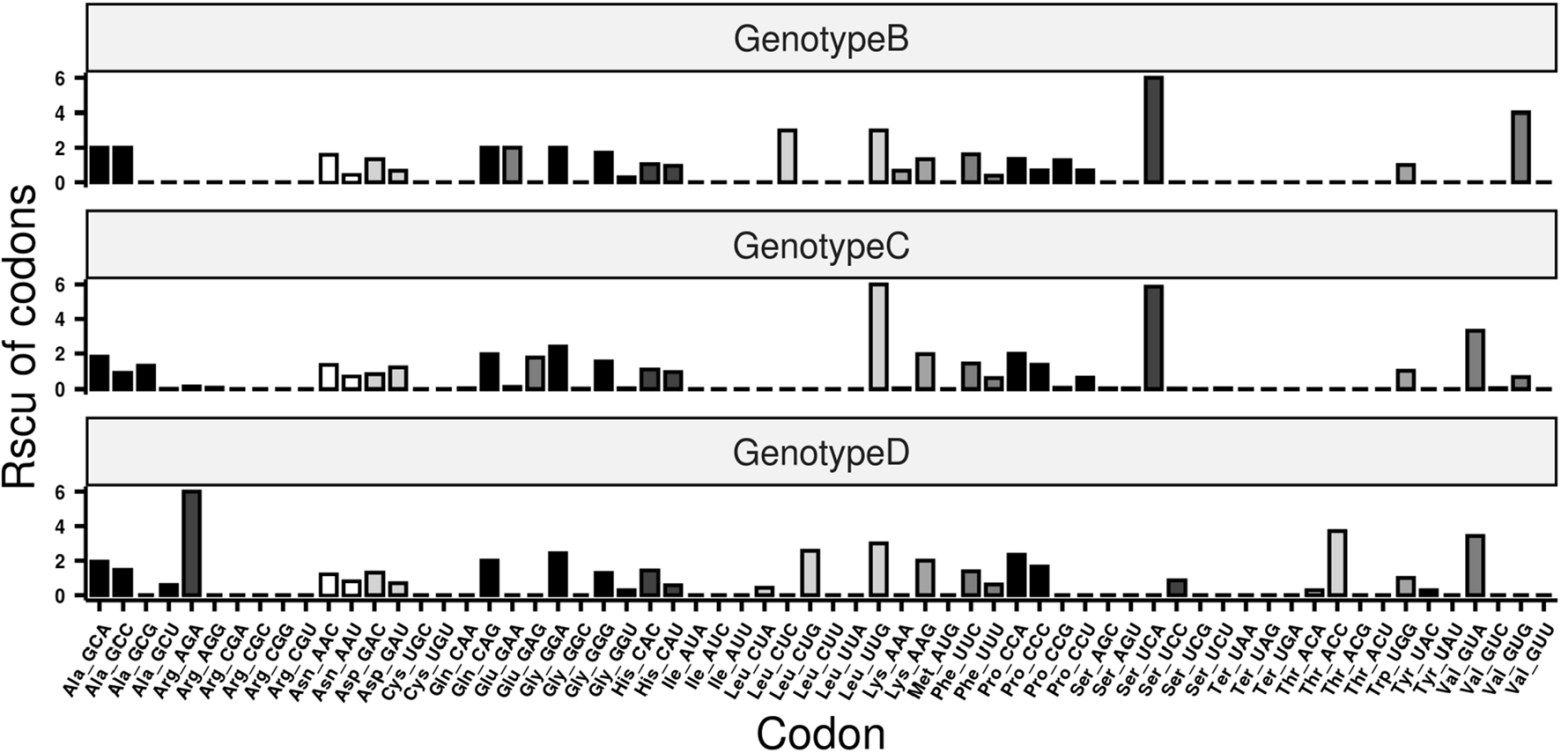
The relative synonymous codon usage (RSCU) values of different codons from the sequences of different genotypes of HBV

**Fig. 6:**
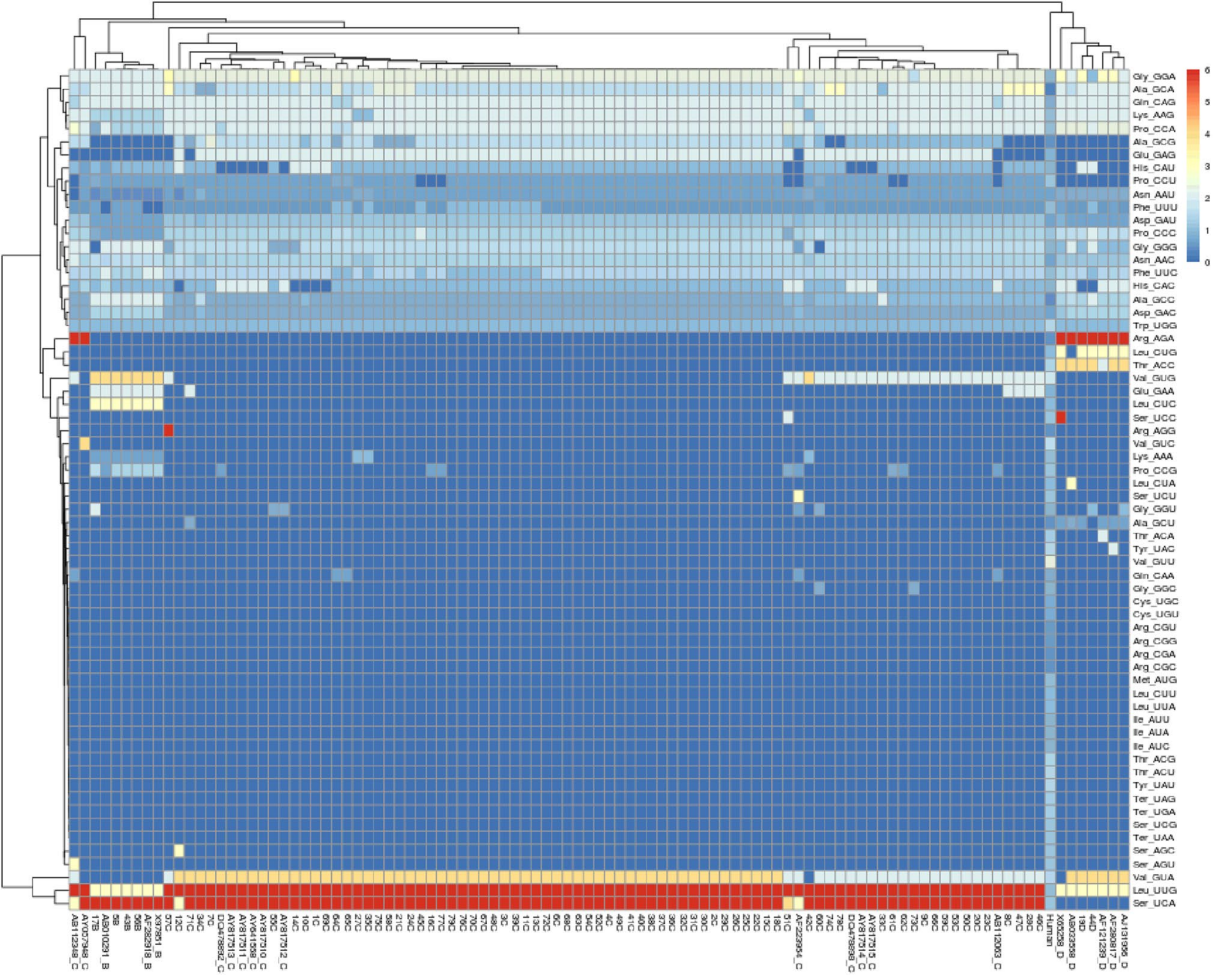
Hierarchical cluster analysis and heat map of the relative synonymous codon usage (RSCU) values of each codon in HBV. Each square in the heat map represents the log ratio of the RSCU value of each codon (in rows) within the HBV genome (in columns). Colors indicate the magnitude of RSCU values: white, RSCU = 1 (no bias in codon usage); blue RSCU < 1; red, RSCU > 1

The RSCU values of each codon within the sequences of 79 HBV isolates were summarized in Supplementary Table S1. Of all of the 64 identified codons, 20 were not present in the 79 HBV sequences. These unused codons were CGA (Arg), CGC (Arg), CGG (Arg), CGU (Arg), UGC (Cys), UGU (Cys), AUA (Ile), AUC (Ile), AUU (Ile), CUU (Leu), UUA (Leu), AUG (Met), UCG (Ser),UAA (Ter), UAG (Ter), UGA (Ter), ACG (Thr), ACU (Thr), UAU (Tyr) and GUU (Val). The remaining 44 codons were used by the HBV genome and were unevenly distributed. In HBV genotype B, 38 codons were unused, 8 codons had mean RSCU < 1, 1 codon had mean RSCU = 1 and 17 codons had mean RSCU > 1. The RSCU values of high-frequency codons in the B genotype ranged between 1 and 6. In the genotype C, 26 codons were unused, 21 had mean RSCU of < 1, one codon had mean RSCU value of 1 while 16 codons had mean RSCU > 1, with the maximum RSCU value of 6. For the genotype D, our results indicated that 26 codons were unused while 10 codons had RSCU values of < 1, 1 codon had mean RSCU of 1 and 17 had mean RSCU of > 1 (Fig. 5). The heatmap and clustering based on the RSCU values (Fig. 6) included 3 types of profiles: preferential codons in red, unused in blue and less-preferred codons in white. The used codons in HBV sequences, as well as the reference strains from Genbank had higher RSCU values than that of human cell, indicating the well adaption of HBV to its host. These results implied that translational selection in nature has an effect on the pattern of synonymous codon usage and the evolutionary pattern of HBV.

**Fig. 7:**
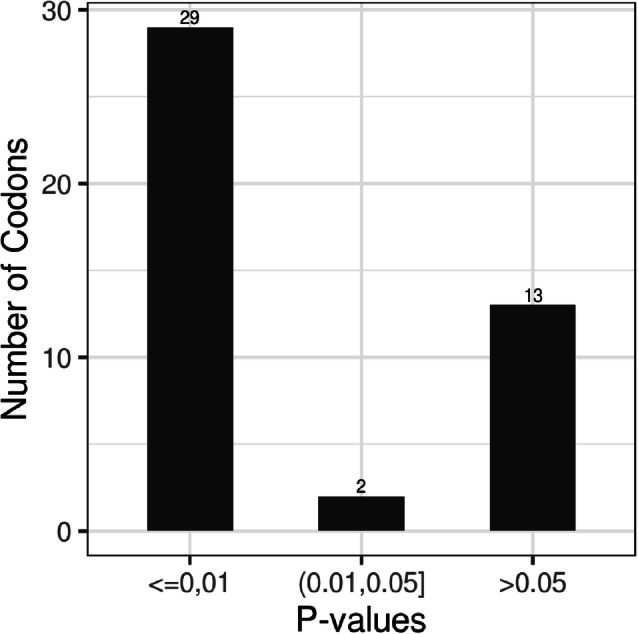
Significantly different codons. The p values were calculated using average RSCU values of each codon in the comparison of the three genotypes

Moreover, to determine the differential usage preference of codons among genotypes, the mean RSCU values were used for one-ANOVA analysis followed by Bonferroni posttest (Fig. 7). The results showed that 31 codons were differentially used among the three genotypes, with 29 of these codons showing a p value of < 0.01 while 2 codons showed p value comprised between 0.01 and 0.05. The remaining 13 codons showed no difference regarding to the usage frequency among HBV genotypes (p > 0.05). These results hinted that the number of differentially used codons between the HBV genotypes was higher than similarly used ones.

### Genetic correlation based on synonymous codon usage in HBV

**Fig. 8:**
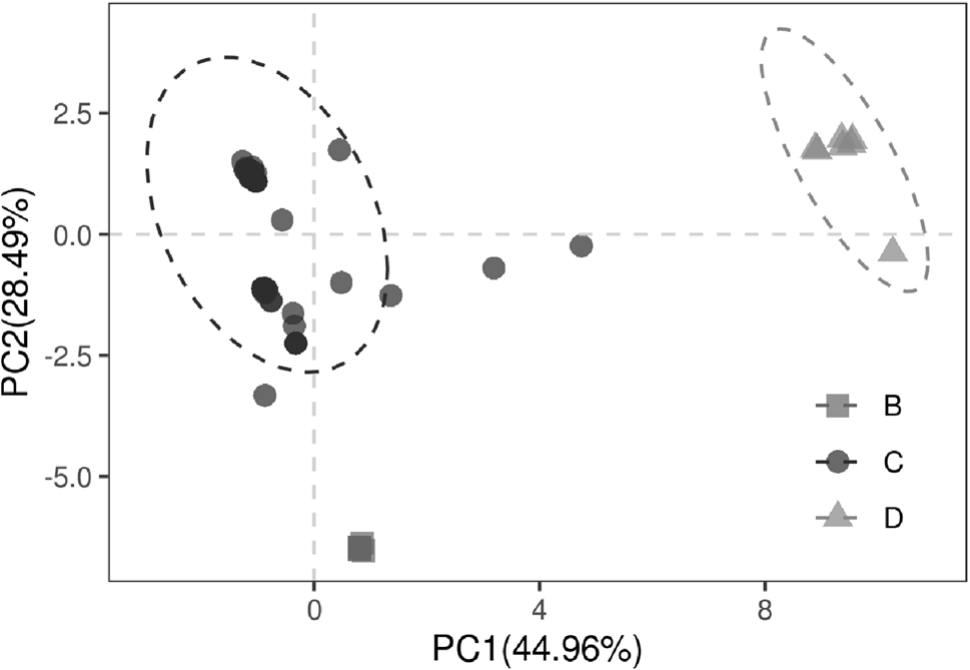
The genetic characteristic of HBV based on different genotypes. Principal component analysis was used for separating the genotypes based on the RSCU values

To explore whether the synonymous codon usage could influence the genotypes of HBV, the principal component analysis (PCA) was performed based on the RSCU values. The PCA detected the first principal component (PC1) which accounted for 44.96% of the total synonymous codon usage variation, and the second principal component (PC2) accounting for 28.49% of total variation (Fig. 8). It can be observed that different HBV genotypes were distinctly separated from each other. Moreover, the genotype B and D showed obviously different genetic characteristics but showed obvious aggregation with genotype C. Thus, the codon usage variation might be one of the factors driving HBV evolution.

### The effect of mutation pressure on codon usage of HBV

We employed three approaches based on codon usage indices, namely a neutrality analysis, an ENC plot, and the ratio of synonymous to non-synonymous substitutions (Ka/Ks), to elucidate the different evolutionary mechanisms operating in the HBV genome.

**Fig. 9:**
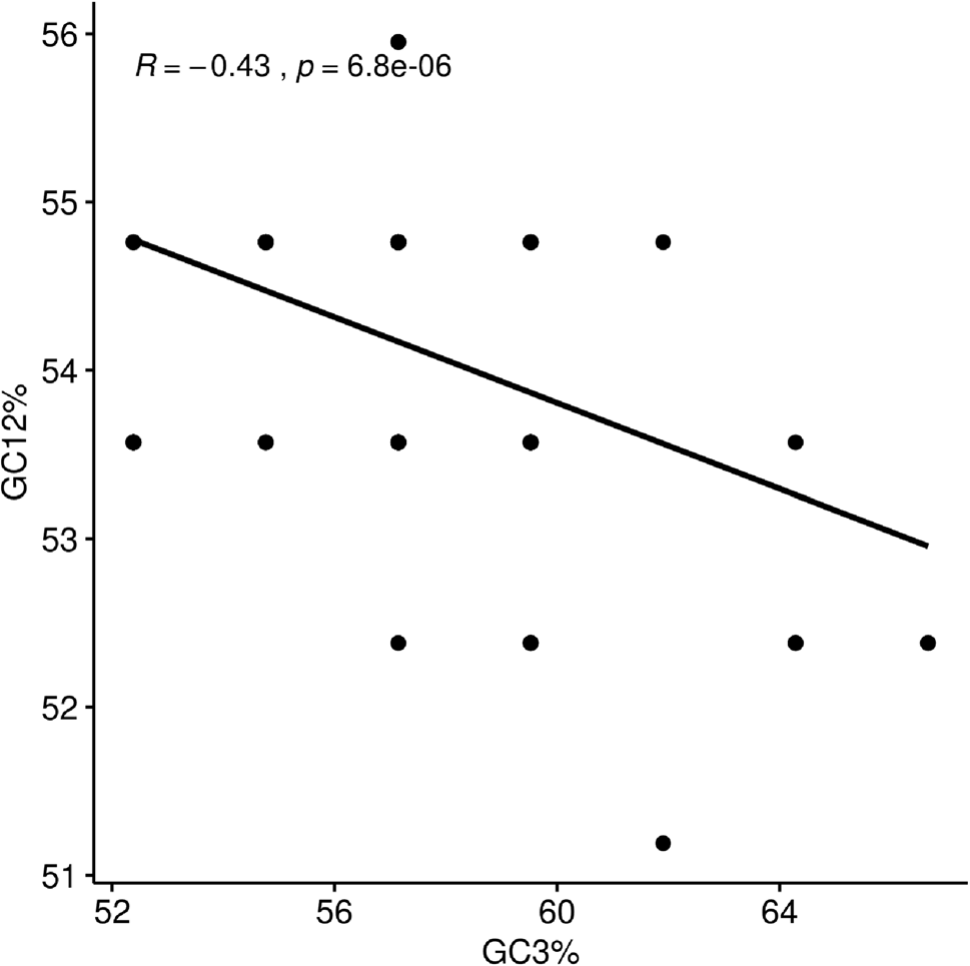
Correlation between GC content at first and second codon position (GC12%) with that at synonymous third codon positions (GC3%)

The neutrality analysis was performed to quantify the mutational pressure. The average values of GC content in the first and second positions (GC12) was 54.15% ± 0.89 while that in the third position (GC3) of codons was 57.26% ± 3.02 (Fig. 9). A significant negative correlation was observed between GC12 and GC3 among all clinical isolates (R = − 0.43, p value = 0.001). Separately, no significant correlation between GC12 and GC3 was found in each genotype. These results suggested that directional mutation bias played a minor role in the evolution of HBV genome.

**Fig. 10:**
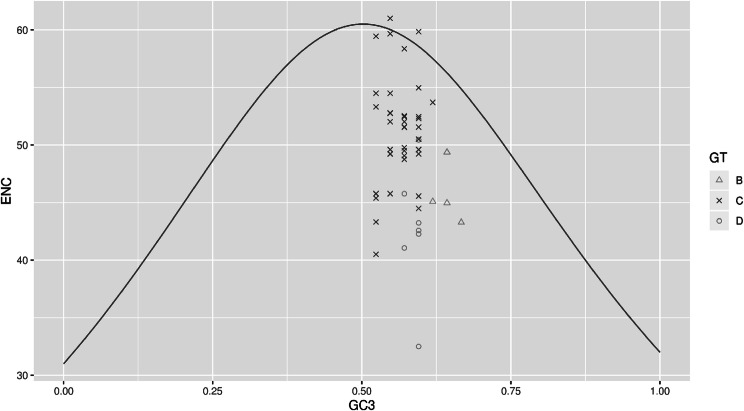
Distribution of the codon usage index, ENc and GC content at synonymous third codon position (GC3%). The curve shows the expected codon usage of GC compositional constraints alone account for CUB

To examine the factors affecting HBV CUB, the ENC values were plotted against the GC3 percentage. As shown in Fig. 10, the points are the actual ENC values and the curve corresponds to the expected ENC values with the only factor of mutation of HBV coding sequences (Fig. 10). It can be observed that the isolates were distributed under and over the expected ENC curve of the C genotype. This implied mutation pressure and factors such as translational selection impact on the CUB of HBV coding sequences for genotype C. The ENC values of genotypes B and D were all under the curve, which suggested that translational selection may be predominant in these genotypes.

**Fig. 11:**
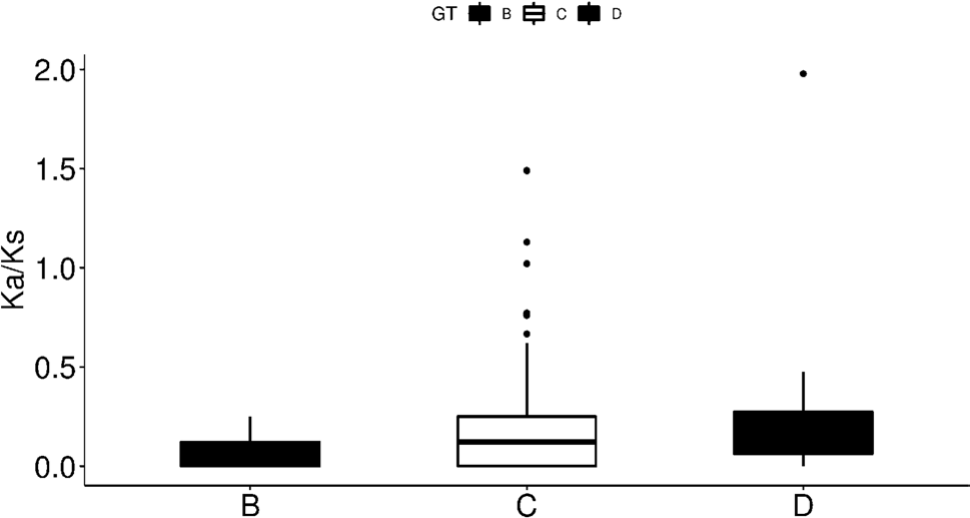
Box-plots of the ratio of synonymous to non-synonymous substitutions (Ka/Ks) in the three HBV genotypes. No significant difference was found (p > 0.05)

The Ka/Ks ratio is a simple measure of selection pressure on codons, which reveals neutrality (Ka/Ks = 1), negative or purifying selection (Ka/Ks < 1), and positive selection (Ka/Ks > 1) (Ma et al. 2011; Woolley et al. 2003). The closer the ratio reaches to 1, the smaller the selection pressure is (Ma et al. 2011). We calculated the Ka/Ks ratio for each genotype, separately. The Ka/Ks ratio ranged from 0 to 0.25 in HBV B genotype, from 0 to 1.98 in the D genotype with only one isolate showing Ka/Ks ≥ 1, and from 0 to 1.49 in C genotype with 3 sequences showing Ka/Ks ≥ 1 (Fig. 11). The majority of these values were significantly lower than 1, implying that the three genotypes are under intense purifying selection. Further comparison of the Ka/Ks ratio between genotypes by one-way ANOVA indicated no significant difference between the Ka/Ks ratio of the three genotypes (Fig. 11), suggesting that there was no difference in the purifying selection among the 3 genotypes.

### Adaptation of HBV to the human genome

**Fig. 12:**
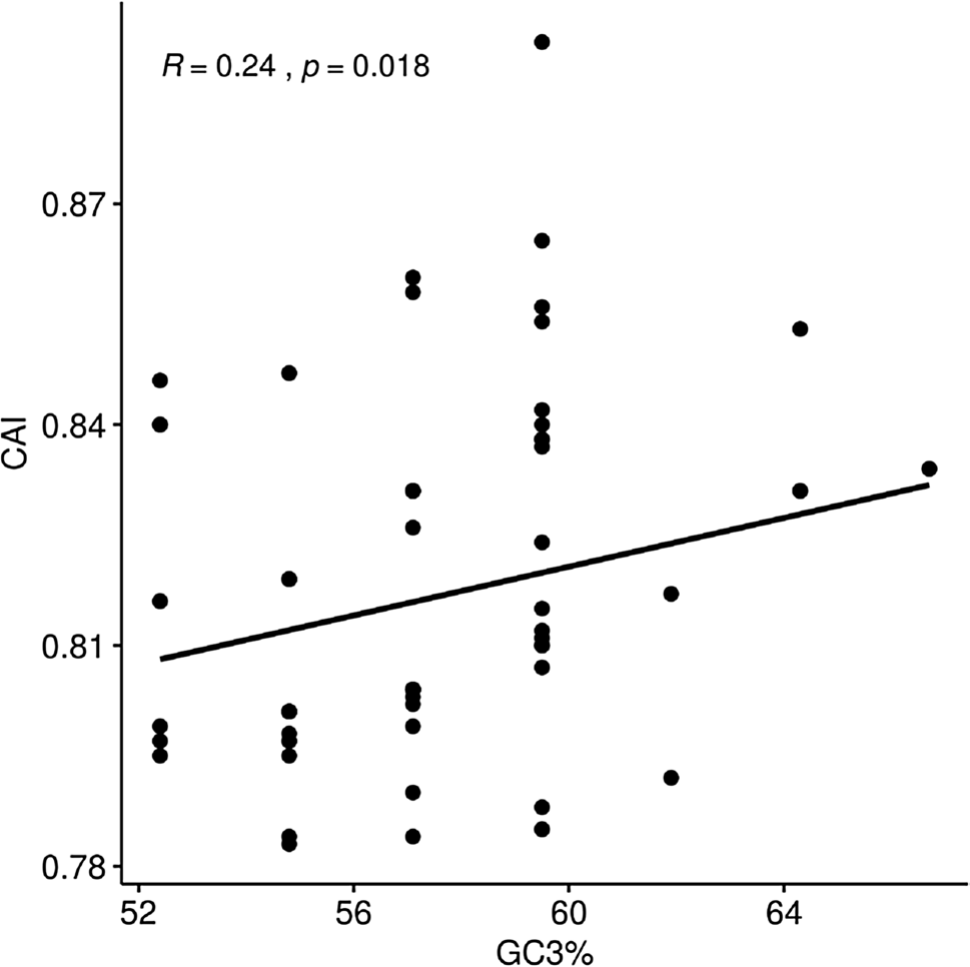
CAI value vs. GC3%. The different genotype points represent the correlation between gene expression and nucleotide composition of HBV coding sequence

The CAI values range from 0 to 1, and high CAI values indicate higher levels of CUB (Subramanian and Rup Sarkar 2015). Codon adaptation index (CAI) analyses were performed to determine the codon usage optimization and adaptation of HBV in relation to its hosts. CAI values for all codons were calculated by reference to the codon usage of *H. sapiens*. We found that, in relation to *H. sapiens*, the CAI values of HBV S small protein-coding regions were in the range of 0.78–0.90 (Fig. 12). This study found a tendency of relatively high CAI values (> 0.5), which disclosed a good adaptability of HBV to its hosts and a low translation pressure (Subramanian and Rup Sarkar 2015). The tendency of high CAI values for *H. sapiens* suggests that selection pressure from *H. sapiens* can affect the codon usage of HBV and that the evolution of codon usage in HBV allows it to use the translation machinery of *H. sapiens* more efficiently. In addition, the results suggested that these differences are related to codon usage preferences. Our results about codon usage preferences were consistent with published works (Ma et al. 2011). Turning to the expression levels of viral product in host cells, the relationship between CAI and GC3% indicates that the various CUBs among B, C, D genotypes exist in the process of evolution of HBV (Fig. 12). This demonstrated that the synonymous codon usage patterns of HBV might play an important role in the optimized expression level of viral product of HBV. Some previous reports pointed out that HBV genotypes have been increasingly associated with differences in virologic and clinical features, such as response to antiviral therapies and severity of liver disease (Enomoto et al. 2006; Kao et al. 2000; Palumbo 2007; Wai et al. 2002).

## Conclusions

This is the first study revealing the codon usage pattern within HBV genotypes. Our study indicated that the CUB of HBV genotypes is low with good adaptability to the human genome. Our findings hinted that mutation bias and mutational pressure were the prevalent factors in shaping HBV codon usage patterns. The present findings have great prospects for elucidating the molecular evolution and functional mechanisms of HBV. The present data will be of clinical importance, especially for studying the pathogenesis of hepatis B and developing treatment drugs.

## Electronic supplementary material

Below is the link to the electronic supplementary material.

Supplementary Table S1: RSCU values of codons in different sequences of HBV (XLS 42 kb)

## Data Availability

All data generated or analyzed during the present study are included in this published article.
